# Gold—Polyoxoborates Nanocomposite Prohibits Adsorption of Bacteriophages on Inner Surfaces of Polypropylene Labware and Protects Samples from Bacterial and Yeast Infections

**DOI:** 10.3390/v13071206

**Published:** 2021-06-23

**Authors:** Mateusz Wdowiak, Enkhlin Ochirbat, Jan Paczesny

**Affiliations:** Institute of Physical Chemistry, Polish Academy of Sciences, Kasprzaka 44/52, 01-224 Warsaw, Poland; mwdowiak@ichf.edu.pl (M.W.); enkhlin1128@gmail.com (E.O.)

**Keywords:** bacteriophages, nanocomposite, surface coatings, antibacterial, antifungal, nanoparticles

## Abstract

Bacteriophages (phages) are a specific type of viruses that infect bacteria. Because of growing antibiotic resistance among bacterial strains, phage-based therapies are becoming more and more attractive. The critical problem is the storage of bacteriophages. Recently, it was found that bacteriophages might adsorb on the surfaces of plastic containers, effectively decreasing the titer of phage suspensions. Here, we showed that a BOA nanocomposite (gold nanoparticles embedded in polyoxoborate matrix) deposited onto the inner walls of the containers stabilizes phage suspensions against uncontrolled adsorption and titer decrease. Additionally, BOA provides antibacterial and antifungal protection. The application of BOA assures safe and sterile means for the storage of bacteriophages.

## 1. Introduction

Bacteriophages, i.e., viruses that infect bacterial cells, were recognized early due to their potential application for medical purposes [1]. In some countries (e.g., Russia, Georgia, Poland, USA), medical institutes use bacteriophages to treat infections caused by bacteria that do not respond to conventional antibiotics [2]. More and more phage-based products reached clinical trials recently for curing various types of disease, including inner ear infections [3], urinary tract infections [4], typhoid [5], systemic multi-drug resistant infections [6], or infected burn wounds (*Phagoburn* project) [7].

Some phages are capable of retaining activity even after exposure to stress factors, such as high temperatures [8], pH [9], and organic solvents [10,11]. However, the resistance to external conditions is not the primary criterion for claiming phages’ value for medical purposes. More often, their virulence, selectivity, ease of manipulation, and/or modification are taken into account, despite phage stability being equally important. Phage stability determining factors were described in the review by Jończyk-Matysiak et al. [12]. The authors focused on the effect of chemical substances, pH, temperature change (freezing, heat), UV-light, and methods of preparation and formulation.

Recently, we found yet another factor affecting the titer of phage suspensions. When stored in plastic containers, phages might adsorb on the surface, significantly reducing the number of active virions in bulk [13]. The effect depends on the number of collisions between virions and the surface. Thus, it is sped up by mixing (introduction of active transport) or by temperature increase. The efficacy of the collisions depends on the hydrophobic/hydrophilic interactions between the surface, water molecules, and virions. When the water wetting angle (WA) of the container insides is higher than a threshold (we found this threshold to be around 95° for T4 phages), it is more favorable to “cover” such a hydrophobic surface with virions. This reduces the system’s overall energy [13]. Such an explanation is in line with findings reported by others related to biomolecules. Biomolecules might assume the conformation in which their hydrophobic parts contact the hydrophobic surface, while more hydrophilic regions are exposed to bulk (and water) [14,15,16]. As a result, the tube having WA higher than the threshold is “unsafe” for phages. The titer of phage suspension might drop by several orders of magnitude in such a container [13].

The importance of phage suspension stability is crucial, especially in phage therapies’ application. In the *Phagoburn* project, improper storage resulted in decreased titer of phages by a thousand-fold within 15 days after the preparation of stocks. Consequently, the patients received a much lower dosage of phages (10^2^ PFU/mL daily) than they were supposed to [7]. In the case of bacteriophages’ encapsulation [17], formulations using emulsions [18], dry-coating formation [19,20], adsorption on the solid surface [21], and freeze-drying [22] were used to increase the stability of virions.

The stabilization of viruses is also important due to the need to protect vaccines [23]. However, as for now, only empirical solutions are available. Freeze-dried preparation [24], immobilization of viral particles [25], and additives, e.g., sucrose [26,27,28], were tested to increase the stability of vaccines. For instance, the preservation of adenovirus for up to 6 weeks at 40 °C was shown employing a sucrose-stabilized liquid formulation [29]. The Stellacci and Vitelli groups describe additives (gold nanoparticles and polyethylene glycol) that improve adenovirus storage by several orders of magnitude at lower concentrations compared to sucrose [30].

Here, we propose a simple countermeasure for uncontrolled adsorption of phages on the inner walls of the containers and, consequently, phage titer decrease. We covered the inside of the containers, in which phage suspensions were stored, with inorganic nanocomposite composed of gold nanoparticles (AuNPs) embedded in the polyoxoborate matrix (BOA) [31]. In addition to stabilizing phage suspension against adsorption, the BOA composite offered protection against bacterial and fungal contaminations. The method of BOA deposition onto standard plastic and glass labware is straightforward and complies with the “green chemistry” approach.

Biocidal nanomaterials usually act via ions’ release [32,33,34] or generation of reactive oxygen species (ROS) [35,36]. Moreover, silver [32,33,34,37,38], copper oxide [34,38,39], titanium dioxide [35,36], and iron-nickel [40] nanoparticles were proved to cause phage deactivation. Gold-based BOA acts via a contact-killing mechanism, i.e., it destabilizes the cell envelope of bacteria and yeasts, and thus it is safe for mammalian cells, which do not possess a cell wall [31]. A different mechanism of action allowed BOA to possess antibacterial and antifungal properties and simultaneously positively affect the stability of phage suspensions.

## 2. Materials and Methods

### 2.1. Chemicals

HAuCl_4_·3H_2_O (99.8%, Aldrich, Saint Louis, Missouri, USA), NaBH_4_ granules (99%, Fluka, Buchs, Switzerland), HCl (analytical grade, POCH, Gliwice, Poland), NaOH (99.8%, POCH, Gliwice, Poland), H_2_SO_4_ (min 95%, POCH, Gliwice, Poland), H_2_O_2_ (30%, Chempur, Piekary Śląskie, Poland) were used as received. Ethanol 95% (POCH, Gliwice, Poland) and chloroform (Chempur, Piekary Śląskie, Poland) used for cleaning were of analytical grade. Ultra-pure water characterized by resistivity 18.2 MΩ∙cm was obtained from the Milli-Q water purification system.

LB-agar contained 15 g/L of agar, 10 g/L of NaCl, 10 g/L of tryptone, and 5 g/L of yeast extract, and it was used as an instant mix (Carl Roth, Karlsruhe, Germany). LB-medium had the same composition except for the lack of 15 g/L of agar (Carl Roth, Karlsruhe, Germany). YPD-agar contained 15 g/L of agar, 20 g/L of bacterial peptone, 20 g/L of glucose, and 10 g/L of yeast extract, and it was used as an instant mix (Carl Roth, Karlsruhe, Germany). YPD-medium had the same composition except for the lack of 15 g/L of agar (Carl Roth, Karlsruhe, Germany). TM buffer was prepared using 10 mM Tris base, 5 μM CaCl_2_, 10 mM MgSO_4_, and distilled water (pH = 7.4). TM buffer components were purchased from Sigma Aldrich (Saint Louis, Missouri, USA). All solutions were sterilized by autoclaving before use. Phosphate buffer (50 mM, pH 7.4) was prepared from NaH_2_PO_4_ and Na_2_HPO_4_ (Carl Roth, Karlsruhe, Germany).

### 2.2. BOA Surface Modification

Gold nanoparticles were prepared according to the procedure described previously [41]. First, an aqueous stock solution of 50 mM gold precursor was prepared by dissolving HAuCl_4_·3H_2_O with the same molar amount of HCl. NaBH_4_ was dissolved with the same molar amount of NaOH (50 mM). A total of 50 µL of HAuCl_4_ solution was added to the Eppendorf tubes containing 1 mL of deionized water. Afterward, 150 µL of NaBH_4_/NaOH were added. Eppendorf tubes containing the reaction mixture were vigorously shaken for 1 min until the color change was observed. The solution turned from light-yellow to brown-orange immediately after adding NaBH_4_/NaOH, then turned to wine-red. Sodium borohydride fulfills two roles: (i) acts as a reductive agent, and (ii) is the source of inorganic oxoborate ligands stabilizing the nanoparticles. The above protocol scales up ideally so one can prepare batches of potentially unlimited volumes. In this study, a 250 mL batch was the maximal volume of AuNPs dispersion prepared.

In this study, the surface modification of the BOA was performed according to the protocol described by us previously [14] with minor changes. In short, vials to be coated were filled with the colloidal AuNPs suspension up to ~2/3 of their maximal volume. Next, the pH of the solution was decreased by the addition of 100 μL of 0.4 M HCl per 1 mL of gold suspension used to obtain optimal deposition. Other acids were tested (e.g., H_2_SO_4_ and H_3_PO_4_); however, the most uniform deposition was obtained by utilizing HCl. Tubes containing the reaction mixture were shaken using a mechanical agitator for one hour to ensure uniform coating of the bottle’s interior (previous procedures proved that the utilization of argon has an insignificant effect on deposition efficiency). Then, the colorless post-reaction liquid was removed, and the bottles were washed with deionized water. If the multiple-layer coating was to be prepared, a fresh portion of AuNPs suspension of identical volume was introduced to already coated bottles, and the above procedure was repeated. In this study, we used single- and triple-coated bottles. Finally, after the last water rinsing, bottles were left to dry overnight on air.

### 2.3. Bacteriophages

The protocol described by others was used for bacteriophage preparation [42]. In short, an early logarithmic culture of *Escherichia coli* BL21 was infected by T4. For MS2 and M13 multiplication, the *Escherichia coli* C3000 strain was used. After lysis, phages were precipitated using polyethylene glycol. The precipitates of phages T4 and M13 were purified by centrifugation and diluted with 1 M NaCl. Then, CsCl gradient centrifugation was applied (Beckman Optima XL70 ultracentrifuge, Ti50 rotor, Brea, CA, USA). T4 and M13 suspensions were dialyzed against a series of TM buffer solutions of decreasing ionic strength. Afterward, 0.2 μg/mL DNase was added to samples with phages T4 and M13 to digest residual DNA remaining in the TM buffer after the whole procedure. In the case of MS2, the lysate was only filtered using 0.22 μM syringe filters.

A droplet plaque counting test was conducted to assess phage activity and virulence. A solution containing 0.4 mL LB medium and 0.5% agar was mixed with 200 μL of refreshed *Escherichia coli* bacteria. Depending on the type of phage, they were *E. coli* BL21 (T4 phage) or *Escherichia coli* C3000 (M13 and MS2 phages). The solution prepared in this way was poured onto a previously prepared petri dish with LB-agar. After the agar with the bacteria solidified, at least eight droplets (5 μL each) of each of the adequately diluted phage suspensions were deposited onto the plate. Subsequently, the plates were incubated at 37 °C for 24 h. After removing the plates from the incubator, the plaques were counted, and the concentration of phages was calculated and expressed in PFU/mL (plaque-forming units). Student’s t-test was performed to evaluate whether observed differences were statistically significant (* *p* < 0.05; ** *p* < 0.01; *** *p* < 0.001).

### 2.4. Bacteria

In antibacterial tests, *Escherichia coli* BL21 (DE3) (obtained from the Institute of Biochemistry and Biophysics in Warsaw, Poland) was used as an example of a Gram-negative strain to estimate the antibacterial properties of BOA. The *Staphylococcus aureus* wild type (WT) (ATCC 33591) strain was used as an example of Gram-positive bacteria. The bacteria were cultured according to standard protocol. First, the single colony from the agar plate was inoculated into LB medium at 37 °C in an orbital shaker. Next, bacterial cultures were diluted with LB medium to reach proper optical density (OD_600_ = 0.5 for *E. coli*, which corresponds to about 6 × 10^7^ CFU/mL; OD_600_ = 0.9 for *S. aureus*, which corresponds to the concentration of bacteria of about 1 × 10^8^ CFU/mL). Afterward, bacterial cultures were diluted by four orders of magnitude in phosphate buffer and incubated at room temperature for 24 h with shaking (400 rpm) in pristine and BOA-modified vials. The initial amount of bacteria in each flask was around 10^3^ to 10^4^ bacteria/mL. At particular time points (after incubation of 0, 1, 3, 6, and 24 h), 100 μL of the solution were transferred on LB-agar plates and spread using glass L-shape spreaders. After overnight incubation at 37 °C, the plate count method was used to determine the viability of bacteria.

Presented results are averaged from at least three independent agar plates for bacterial experiments. Student’s t-test was performed to evaluate whether observed differences were statistically significant (* *p* < 0.05; ** *p* < 0.01; *** *p* < 0.001).

### 2.5. Yeasts

The *Saccharomyces cerevisiae* wild type (WT) strain (obtained from the Institute of Biochemistry and Biophysics in Warsaw, Poland) was used as an example of fungi (yeast) to estimate the antifungal properties of BOA. The yeasts were cultured according to standard protocol. A single yeast colony was inoculated into YPD-medium. Next, the culture was diluted with YPD medium and incubated to reach optical density OD_600_ = 1.25, which corresponds to the concentration of about 2 × 10^7^ cells/mL. Afterward, yeast cultures were diluted by three orders of magnitude in phosphate buffer for 24 h with shaking (400 rpm). The starting amount of yeasts in each flask was around 10^3^ cells/mL.

At particular time points (after incubation of 0 h, 1 h, 3 h, 6 h, and 24 h) 100 μL of the solution were transferred on YPD-agar plates and spread. After overnight incubation at 37 °C, the plate count method was used to determine the viability of yeasts. Presented results are averaged from at least three independent agar plates for yeast experiments. Student’s t-test was performed to evaluate whether observed differences were statistically significant (* *p* < 0.05; ** *p* < 0.01; *** *p* < 0.001).

### 2.6. Instrumentation

Scanning electron microscopy observations were executed using the FEI Nova NanoSEM 450 (Hillsboro, OR, USA) with an accelerating voltage of 5 kV to 10 kV under high vacuum. The microscope was equipped with an EDS (EDX) (Energy Dispersive X-ray Spectroscopy) spectrometer, allowing for analysis of the chemical composition in micro areas.

The surface of polypropylene samples was sputtered using Quorum Technologies LTD. K550X equipped Quorum Technologies LTD. K150X thickness monitor. Sputtering was done at 10 mA for 600 s to obtain a layer of a thickness of around 10 nm. This thin gold layer was necessary to visualize the surface using SEM, as polypropylene is not conductive and charges upon exposition to the beam of electrons.

Dynamic light scattering (DLS) measurements were performed using a Zetasizer Nano ZS apparatus (Malvern Instruments Ltd., Malvern, UK) equipped with a dynamic light scattering (DLS) module (He−Ne laser 633 nm, max 4 mW).

UV-Vis absorbance spectra were recorded on a Thermo Scientific Evolution 201 UV-vis spectrophotometer in the spectral range from 350 to 750 nm in 1 cm optical path glass cuvettes.

## 3. Results and Discussion

Our recent publication showed that virions might “disappear” from the suspension, thus decreasing the apparent titer of the phage samples. We found that the “missing” virions are adsorbed onto the surface of plastic labware. The Eppendorf-type and Falcon-type polypropylene surface can accommodate from around 10^8^ PFU/mL to around 10^10^ PFU/mLT4 virions from the suspension [13]. The magnitude of this effect varies for tubes purchased from various vendors and also between batches provided by a single vendor.

**Figure 1:**
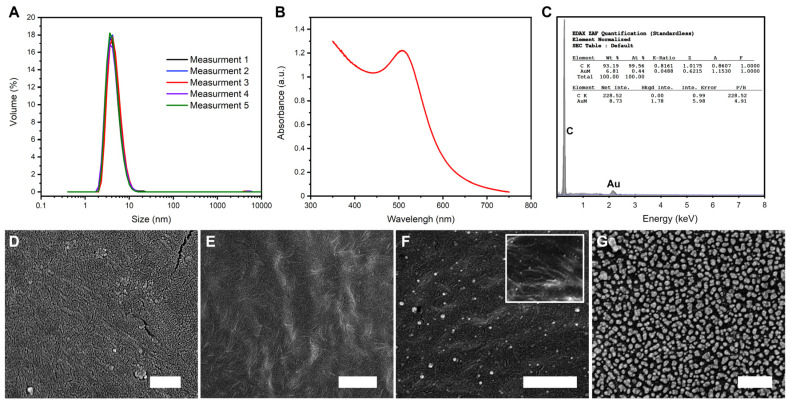
(**A**) DLS measurements show the hydrodynamic diameter of the initial AuNPs equal to 4.5 ± 1 nm. (**B**) UV-Vis measurement of the initial AuNPs. (**C**) EDX spectrum of BOA deposited onto the polypropylene surface, proving gold presence within the coating. SEM pictures of (**D**) pristine polypropylene vial used in the study; (**E**) pristine vial after 24 h incubation with M13 phages. Visible fiber-like features are virions. (**F**) The inner surface of the vial after single deposition of BOA, and (**G**) triple deposition of BOA. The inset in (**C**) shows the same surface as the main picture in (**C**), but without an additional thin layer of gold-sputtered to facilitate SEM observations. Scale bars correspond to 500 nm.

As a solution for this problem, we propose the utilization of a BOA (B—boron, O—oxygen, and A—gold (from Latin: aurum, Au)) nanocomposite [31]. First, “naked” gold nanoparticles (i.e., without an organic ligand shell) are produced in a procedure described previously [41]. In this method, the used reducing agent (sodium borohydride) becomes a source of oxoborates ions stabilizing the colloidal solution. At low concentrations (below 25 mM), both boric acid and borate typically exist as monomers. The amount of the planar BO_3_ and the tetrahedral BO_4_ moieties depends on the pH [43]. In the solutions of a higher concentration, the equilibrium is established between a unionized form of boric acid and polynuclear complexes B_3_O_3_(OH)_4_^−^, B_4_O_5_(OH)_4_^2−^, B_3_O_3_(OH)_5_^2−^, B_5_O_6_(OH)_4_^−^, and B(OH)_4_^−^. The change in pH leads to condensation and the formation of more complex polyanions. In the acidic pH, planar nets stacked together are formed [44]. In the presence of gold nanoparticles (around 4.5 ± 1 nm in diameter as revealed by DLS and confirmed based on the maximum of the UV-Vis absorbance; see Figure 1A,B), the metallic cores become embedded in the polyoxoborate matrix. This leads to the formation of a nanocomposite coating (BOA). BOA building blocks are amphiphilic. The amphiphilicity is related to charge delocalization and the reduction in the formal charge of polymerized oxoborate anions (hydrophobic properties), and the presence of accessible OH groups at the edges (hydrophilic properties). Because of this property, BOA can be successfully deposited on hydrophilic (often employing condensation with surface OH groups) and hydrophobic surfaces [31].

The concentration of gold nanoparticles suspension limited the amount of deposited BOA. To increase the surface coverage, we performed multiple deposition processes. Scanning electron microscopy examinations clearly showed the difference between single (Figure 1F) and triple (Figure 1G) BOA deposition. After single deposition, sparsely distributed bright objects of the size of around 30 to 50 nm (significantly larger compared to initial nanoparticles of the diameter of approximately 4.5 nm) were visible at the surface (Figure 1F). A densely packed and uniform layer of similar bright objects was visible after multiple deposition processes (Figure 1G). In the case of pristine vials (Figure 1D) only cracks in the sputtered gold layer were observed. The inset in Figure 1F shows the surface of the same sample as the main picture but with lower resolution, as this sample was not sputtered. Similar morphological features were visible in sputtered and non-sputtered samples, which excluded the possibility that the discussed bright objects are artifacts coming from sample post-processing. Elemental analysis of BOA coating, executed using EDX (energy-dispersive X-ray) spectroscopy, confirmed that the mentioned bright objects were loaded with gold, proving their identity as BOA (Figure 1C).

To show the efficiency of BOA in the stabilization of phage suspensions, we studied three phages, namely MS2, M13, and T4. These represent three major types of phages: symmetrical, filamentous, and tailed phages, respectively. The MS2 bacteriophage, belonging to *Fiersviridae*, has an icosahedral structure, and its genetic material is (+)ssRNA. It has a diameter of about 27 nm and has one of the smallest known viral genomes so far (3569 nucleotides) [45]. MS2 is often regarded as a good model for studies on eukaryotic (also pathogenic) viruses [46,47,48]. M13 is a member of the filamentous bacteriophage family (*Inoviridae*). It is a virus “nanofiber”: 880 nm in length and 6.6 nm in diameter [49]. Its genome is in ssDNA form. M13 causes chronic infections, i.e., progeny virions are secreted continuously, without disrupting the host cells [50]. M13 is often used in the phage display method [51]. T4 belongs to *Caudovirales*—tailed phages. It is the most abundant order of bacteriophages. Ackermann estimated that more than 90% of all phages are tailed [52]. It is estimated that there are more bacteriophage virions at any given moment (around 10^31^) than any other organisms, including bacteria, combined [53]. However, we believe that the selection of phages used in this study represents the majority of possible applications.

**Figure 2:**
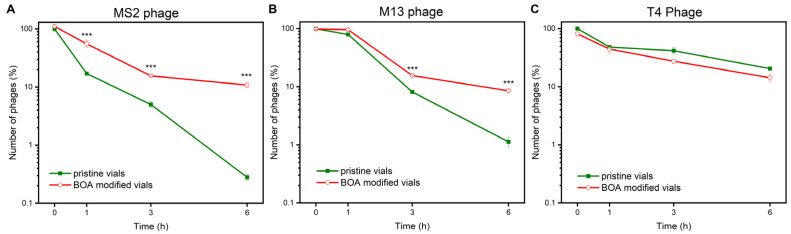
Comparison of the number of bacteriophages presented as a percent of concentration at 0 h. The initial concentrations were (**A**) 3.75 × 10^3^ ± 3.74 × 10^2^ PFU/mL for MS2, (**B**) 5.08 × 10^3^ ± 1.60 × 10^2^ PFU/mL for M13, and (**C**) 6.60 × 10^5^ ± 5.01 × 10^4^ PFU/mL for T4. The visible decrease was visible in pristine vials in M13 and MS2 but not in T4. BOA showed the statistically significant protection against the phage titer decrease in the case of M13 and MS2 and did not show any adverse effect on T4 suspensions (*** *p* < 0.001).

First, we incubated phages in polypropylene containers with mixing. Mechanical agitation increased the number of collisions between virions and the inner walls of the container, effectively facilitating the adsorption. For MS2 and M13, we found a significant decrease (>99%) of phage titer after six hours of mixing in the pristine, non-modified vials (Figure 2). For T4, a much smaller decrease was observed.

It was proved before that the adsorption of phages is governed by the WA [13]. Above the threshold value, phages adsorb onto plastic surfaces. SEM observation proved that M13 virions were, in fact, present at the surface of non-modified vials after 24 h incubation (Figure 1E). For T4 phages, this threshold was found to be around 95° [13]. Here, the wetting angle of the pristine vials was around 93.2° ± 0.2°. This is why we did not observe a dramatic decrease in T4 titer in pristine vials.

Next, we aimed to protect MS2 and M13 suspensions from uncontrolled “disappearing” from the solution due to adsorption on the inner walls of the container. For this, the BOA nanocomposite was deposited onto the inner walls of containers in which phage suspensions were stored. The process of BOA deposition complies with the “green chemistry” approach. First, this method is water-based. What is more important, upon deposition of BOA, the suspension of gold nanoparticles changed color from wine red to transparent. This proved that the vast majority of gold nanoparticles adhered to the surface, limiting the amount of waste generated [31].

Upon single deposition of BOA, no phage protection was observed (data not shown). We observed significant stabilization of MS2 and M13 suspensions upon triple deposition of BOA onto the inner surface of the containers (Figure 2A,B). We found that the saturation of the surface with BOA reduced the wetting angle (WA) from around 93.2° ± 0.2° to around 83.8° ± 1.3°. The titers of MS2 and M13 suspensions were more than an order of magnitude higher compared to the pristine vials after 6 h incubation with mixing. BOA prohibited the adsorption of virions on polypropylene surfaces. It changed the character of the vials from “unsafe” to “safe” by decreasing WA (as proved in our previous study in the case of plasma treatment [13]).

There was no statistical difference in the case of T4 phages between BOA-covered and pristine polypropylene containers (Figure 2C), which both caused only a small decrease in the T4 phage titer. This was in line with the measured WA of the pristine vials being below the threshold found for T4. This result also proved that BOA was safe for phages.

We hypothesized that structural differences caused the difference between the titer decrease between MS2, M13, and T4. Two aspects need to be analyzed: morphology and electrical properties. At the studied pH (7.4), the zeta potential of MS2 is around −40 mV [54], and of M13 around −15 mV [55]. These phages lack distinctive morphological features. On the contrary, T4 phages have a complex structure and a significant dipole moment, reaching hundreds of thousands of Debays, depending on the conformation of fibers [56,57]. In addition, the fibers bear a large positive charge, which facilitates the initial recognition of the bacterial cell. The experiment at higher ionic strength (100 mM buffer instead of 10 mM buffer) did not show any significant differences in results (data not shown). In such a case, the electrostatic interactions were screened effectively by the additional ions. This suggested that observed differences in behavior between MS2, M13, and T4 were due to the specific structure of tailed T4 that influenced the WA threshold. The hydrophobic-hydrophilic balance seems more critical than electrostatic interactions in governing the adsorption process.

We excluded leachables (chemicals released from the plastic into the buffer) as a cause of phage titer decrease. Leachables are usually additives used for stabilizing and modifying end-product properties. Such additives are not chemically bound but form a solid mixture, from which they might be released [58]. Some additives, e.g., plasticizers, slip agents, or biocides, are potentially toxic [59]. We performed the experiment in which the TM buffer was first incubated (without phages) in pristine and BOA-covered (three depositions) containers for 24 h. From now, we use the term TM_P and TM_P_BOA to describe buffers potentially containing leachables. Next, TM, TM_P, and TM_P_BOA were used to prepare phage suspensions (final contents of TM_P and TM_P_BOA were not lower than 99.9%), which were later incubated in “safe” vials. These “safe” vials were Eppendorf tubes, onto which phages did not adsorb upon storage, heating, or mixing [13]. We did not find any difference between phage titers in TM, TM_P, and TM_P_BOA in all studied cases (MS2, M13, and T4). This proved that any potential leachables did not cause the decrease in phage titers showed in Figure 2, but the interaction between virions and the surface caused the phenomena.

In addition, we deposited BOA on the inner surfaces of “safe” polypropylene vials (Eppendorf type tubes, onto which none of the studied phages adsorb). The titer change upon 6 h incubation with mixing was minuscule and similar in the case of both pristine “safe” tubes and BOA-covered tubes. This proved that BOA has no adverse effect on phages and can only protect the phage suspensions against uncontrolled adsorption. In the long run, the increased stability of phage suspensions, and additional protection from infections, would benefit the quality of phage-related research. This would also positively impact the phage therapies, protecting from uncontrolled changes in phage titer and inefficiency of treatment related to this issue.

**Figure 3:**
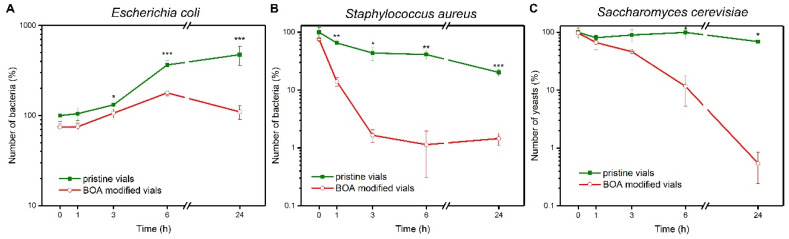
Comparison of growth-rate of (**A**) *Escherichia coli* BL21 (DE3) (initial concentration of 1.78 × 10^3^ ± 2.38 × 10^2^ CFU/mL), (**B**) *Staphylococcus aureus* WT (initial concentration of 1.53 × 10^4^ ± 3.28 × 10^3^ CFU/mL), and (**C**) *Saccharomyces cerevisiae* WT (initial concentration of 8.25 × 10^4^ ± 1.84 × 10^4^ CFU/mL) in pristine vials and BOA-modified vials. In all cases, BOA caused a decrease in the number of viable cells compared to pristine vials (* *p* < 0.05; ** *p* < 0.01; *** *p* < 0.001).

Besides stabilizing phage suspensions, BOA also offers protection against bacterial and yeast infection. We incubated Gram-negative (*Escherichia coli*) and Gram-positive (*Staphylococcus aureus*) bacteria in pristine and BOA-modified polypropylene containers. Only a single BOA deposition was enough to observe a significant effect on bacteria. In Gram-positive *S. aureus,* a considerable decrease (around 2log) was observed in BOA-modified vials (Figure 3B). The number of bacterial cells changed only slightly in the non-modified containers. Experiments on *E. coli* repeatedly showed an increase in the number of bacterial cells in non-modified vials (Figure 3A). After 24 h, the number of cells in the suspension increases around ten times. This phenomenon was explained by Garvie in the 1950s, who found that “*E. coli* will grow when cell suspensions are inoculated into phosphate buffer” [60]. In fact, only three cell division cycles were needed to increase the number of cells eight-fold, i.e., close to the observed increase. In the BOA-covered vials, the number of *E. coli* cells remained approximately still throughout the time span of the experiment. There may be two reasons for this phenomenon: (i) BOA effectively inhibits replication, or (ii) it kills bacteria at the same pace as they replicate. After 24 h, the number of bacteria in the control sample was approximately ten times higher than in BOA-protected vials. The difference in the magnitude of the effect of BOA between Gram-positive and Gram-negative is in line with previous findings, showing that Gram-positive bacteria are much more sensitive to physical and chemical factors compared to Gram-negative bacteria [61].

Antifungal properties of BOA are equally important, as one of the most common contaminants in laboratory practice and in the food industry are yeasts [62,63]. For *S. cerevisiae* WT (Figure 3)*,* we observed a decrease in the number of yeast cells of around 2 log upon utilization of BOA-covered vials (single deposition process) compared to pristine polypropylene vials.

## 4. Conclusions

The quality of labware is an uncontrollable variable that might affect the reproducibility of phage-related studies or the efficacy of phage therapies. Our previous publication showed that virions adsorb on the inner walls of plastic containers when the water wetting angle exceeds the threshold value. Here, we successfully utilized the BOA composite for bacteriophage storage. The application of the BOA composite not only decreased the uncontrolled adsorption of virions at the plastic surfaces but also provided antibacterial and antifungal protection. The process of BOA deposition is straightforward and complies with the requirements of “green chemistry”, as the protocol is water-based and waste limiting. These findings are a step towards increased reproducibility of phage-related research. BOA might also be implemented for the storage of phage cocktails for phage-therapies and biocontrol applications.

## Data Availability

The datasets obtained and analyzed during the current study are available from the corresponding author on reasonable request.
